# The genome sequence of Svensson’s copper underwing, *Amphipyra berbera *Rungs, 1949

**DOI:** 10.12688/wellcomeopenres.17350.1

**Published:** 2021-11-18

**Authors:** Douglas Boyes, Liam M. Crowley, Peter W.H. Holland, University of Oxford and Wytham Woods Genome Acquisition Lab, Darwin Tree of Life Barcoding collective, Wellcome Sanger Institute Tree of Life programme, Wellcome Sanger Institute Scientific Operations: DNA Pipelines collective, Tree of Life Core Informatics collective, Darwin Tree of Life Consortium

**Affiliations:** 1 UK Centre for Ecology & Hydrology, Wallingford, UK; 2 Department of Zoology, University of Oxford, Oxford, UK

**Keywords:** Amphipyra berbera, Svensson’s copper underwing, genome sequence, chromosomal

## Abstract

We present a genome assembly from an individual male *Amphipyra berbera* (Svensson’s copper underwing; Arthropoda; Insecta; Lepidoptera; Noctuidae). The genome sequence is 582 megabases in span. The majority (99.97%) of the assembly is scaffolded into 31 chromosomal pseudomolecules, with the Z sex chromosome assembled.

## Species taxonomy

Eukaryota; Metazoa; Ecdysozoa; Arthropoda; Hexapoda; Insecta; Pterygota; Neoptera; Endopterygota; Lepidoptera; Glossata; Ditrysia; Noctuoidea; Noctuidae; Amphipyrinae; Amphipyra; *Amphipyra berbera* Rungs, 1949 (NCBI:txid987877).

## Background

*Amphipyra berbera* (Svensonn’s copper underwing) is a large noctuid moth with broad, brown forewings patterned with pale zigzags and hindwings suffused with copper brown. The moth has been recorded across much of Eurasia and North Africa; in the UK it is common across England and Wales with scattered records from Scotland. Svensonn’s copper underwing is very similar morphologically to *A. pyramidea* (copper underwing) and was initially considered a subspecies *A. pyramidea berbera* ( Rungs, 1949) until recognised as a separate species in 1968 ( Fletcher, 1968). Adults can be distinguished by the extent of the copper colouration on the underside of the hindwing and larvae can be separated by the colour of the dorsal point on abdominal segment eight. The status of *A. berbera* as a distinct species is supported by mitochondrial COI barcode data ( Chen *et al.*, 2013). Larvae of *A. berbera* feed on the leaves of several deciduous trees, frequently oak ( *Quercus*). In the UK, adults fly from June to September and are attracted to sweet substances including tree sap. The overwintering stage is as an ovum and pupation occurs underground.

## Genome sequence report

**Figure 1.**
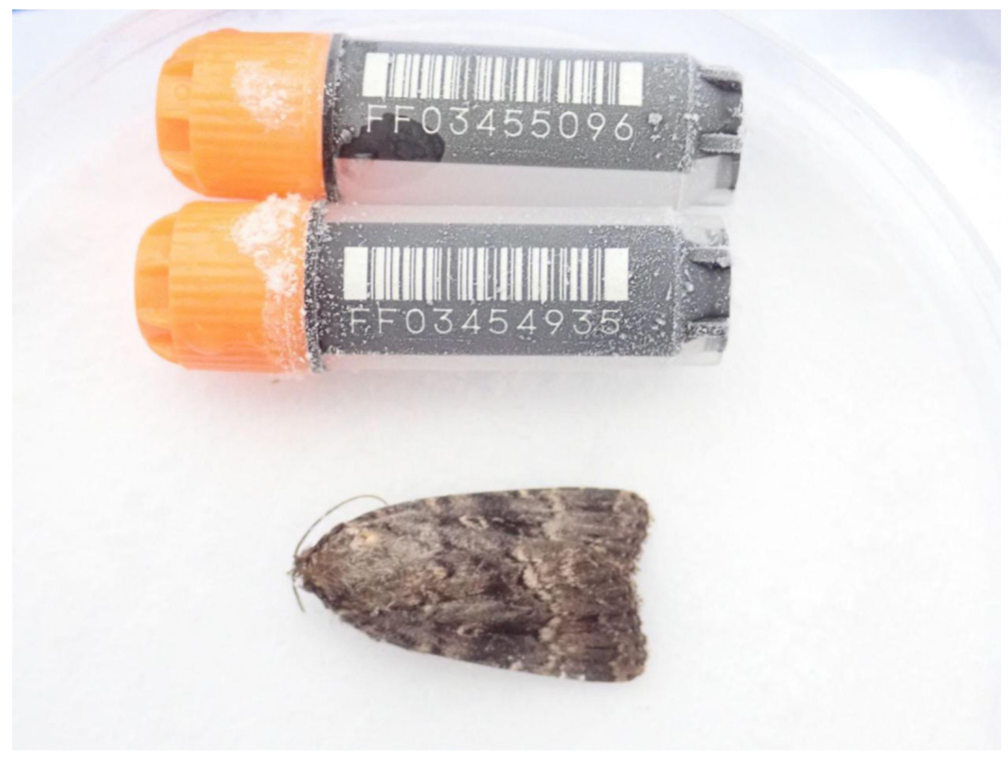
An image of the sequenced specimen, ilAmpBerb1, captured immediately prior to processing and preservation.

The genome was sequenced from one male *A. berbera* ( Figure 1) collected from Wytham Woods, Oxfordshire (biological vice-county: Berkshire), UK (latitude 51.772, longitude -1.338). A total of 41-fold coverage in Pacific Biosciences single-molecule long reads and 71-fold coverage in 10X Genomics read clouds were generated. Primary assembly contigs were scaffolded with chromosome conformation Hi-C data. Manual assembly curation corrected 8 missing/misjoins, reducing the assembly length by 0.01% and the scaffold number by 15.38%.

**Figure 2.**
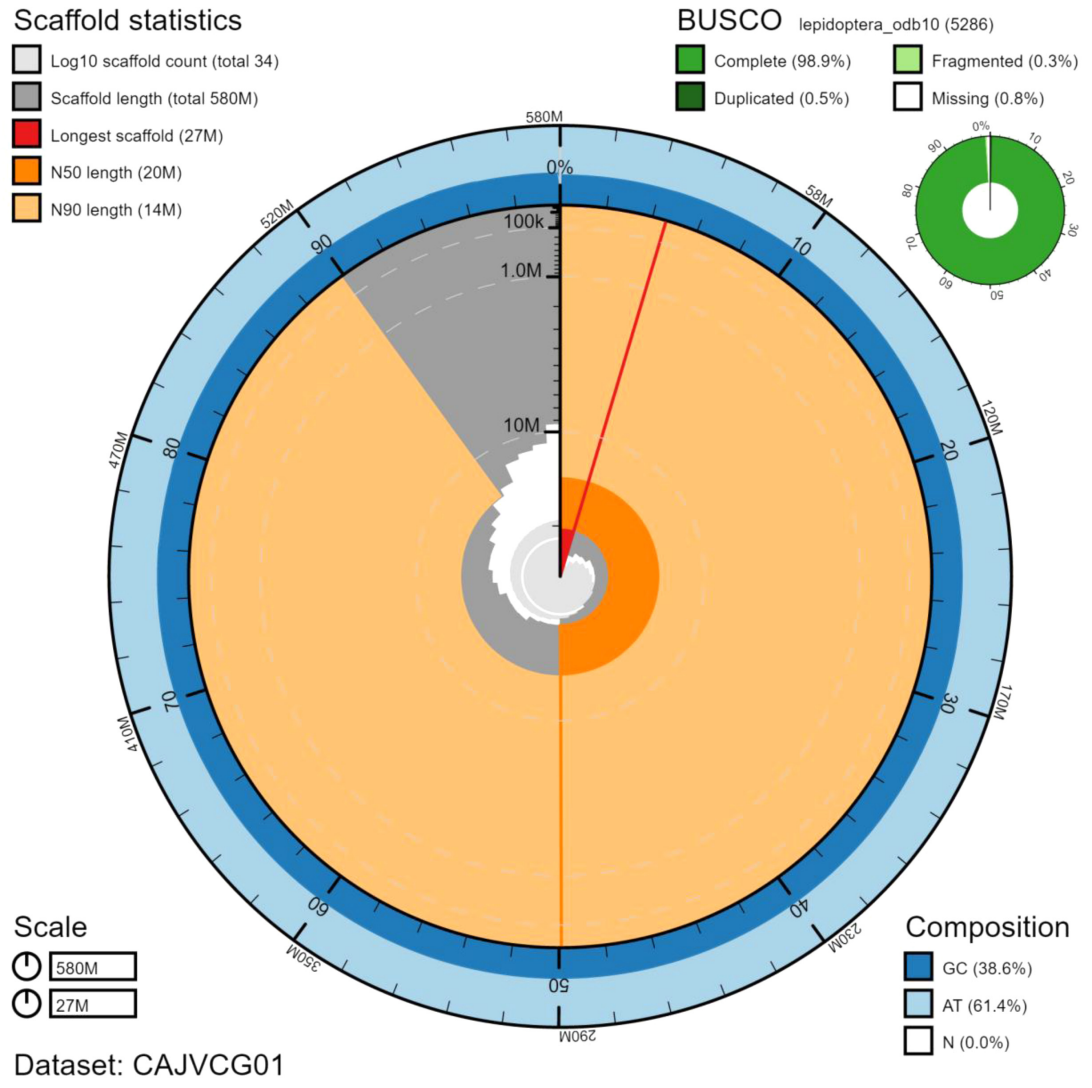
Genome assembly of *Amphipyra berbera*, ilAmpBerb1.1: metrics. The BlobToolKit Snailplot shows N50 metrics and BUSCO gene completeness. The main plot is divided into 1,000 size-ordered bins around the circumference with each bin representing 0.1% of the 582,329,976 bp assembly. The distribution of scaffold lengths is shown in dark grey with the plot radius scaled to the longest scaffold present in the assembly (26,789,203 bp, shown in red). Orange and pale-orange arcs show the N50 and N90 scaffold lengths (20,126,316 and 14,403,444 bp), respectively. The pale grey spiral shows the cumulative scaffold count on a log scale with white scale lines showing successive orders of magnitude. The blue and pale-blue area around the outside of the plot shows the distribution of GC, AT and N percentages in the same bins as the inner plot. A summary of complete, fragmented, duplicated and missing BUSCO genes in the lepidoptera_odb10 set is shown in the top right. An interactive version of this figure is available at https://blobtoolkit.genomehubs.org/view/ilAmpBerb1.1/dataset/CAJVCG01/snail.

The final assembly has a total length of 582 Mb in 33 sequence scaffolds with a scaffold N50 of 20.1 Mb ( Table 1). The majority, 99.97%, of the assembly sequence was assigned to 31 chromosomal-level scaffolds, representing 30 autosomes (numbered by sequence length), and the Z sex chromosome ( Figure 2– Figure 5; Table 2). The assembly has a BUSCO v5.1.2 ( Manni *et al.*, 2021) completeness of 98.9% (single 98.5%, duplicated 0.5%) using the lepidoptera_odb10 reference set. While not fully phased, the assembly deposited is of one haplotype. Contigs corresponding to the second haplotype have also been deposited.

**Table 1.** Genome data for *Amphipyra berbera*, ilAmpBerb1.1.

*Project accession data*	
Assembly identifier	ilAmpBerb1.1
Species	*Amphipyra berbera*
Specimen	ilAmpBerb1
NCBI taxonomy ID	NCBI:txid987877
BioProject	PRJEB45130
BioSample ID	SAMEA7701493
Isolate information	Male, abdomen (genome assembly), head/thorax (Hi-C)
*Raw data accessions*	
PacificBiosciences SEQUEL II	ERR6436378
10X Genomics Illumina	ERR6054818-ERR6054821
Hi-C Illumina	ERR6054817
*Genome assembly*	
Assembly accession	GCA_910594945.1
Accession of alternate haplotype	GCA_910595045.1
Span (Mb)	582
Number of contigs	39
Contig N50 length (Mb)	19.9
Number of scaffolds	33
Scaffold N50 length (Mb)	20.1
Longest scaffold (Mb)	23.5
BUSCO * genome score	C:98.9%[S:98.5%,D:0.5%],F:0. 3%,M:0.8%,n:5286

*BUSCO scores based on the lepidoptera_odb10 BUSCO set using v5.1.2. C= complete [S= single copy, D=duplicated], F=fragmented, M=missing, n=number of orthologues in comparison. A full set of BUSCO scores is available at https://blobtoolkit.genomehubs.org/view/ilAmpBerb1.1/dataset/CAJVCG01/busco.

**Figure 3.**
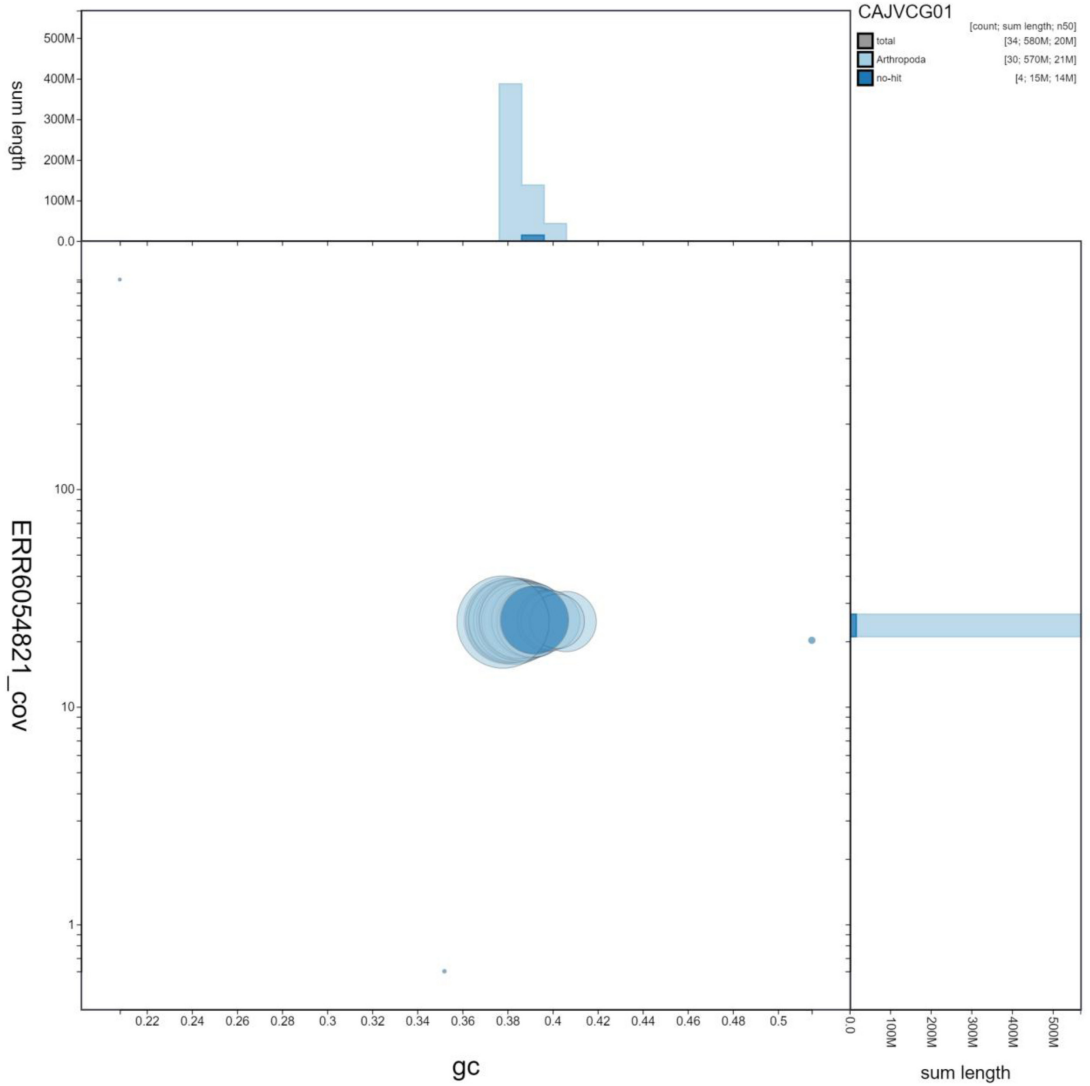
Genome assembly of *Amphipyra berbera*, ilAmpBerb1.1: GC coverage. BlobToolKit GC-coverage plot. Scaffolds are coloured by phylum. Circles are sized in proportion to scaffold length Histograms show the distribution of scaffold length sum along each axis. An interactive version of this figure is available at https://blobtoolkit.genomehubs.org/view/ilAmpBerb1.1/dataset/CAJVCG01/blob.

**Figure 4.**
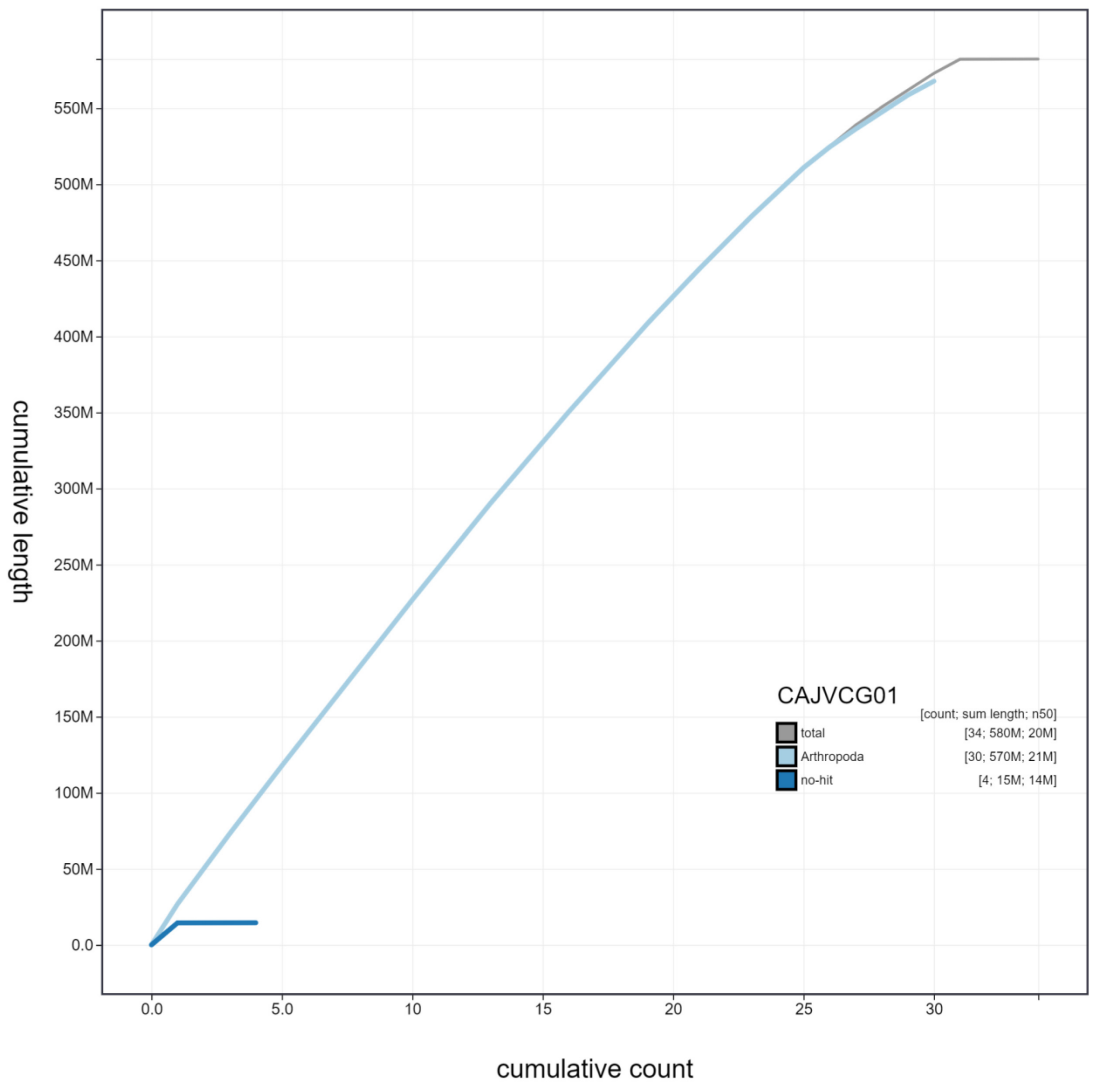
Genome assembly of *Amphipyra berbera*, ilAmpBerb1.1: cumulative sequence. BlobToolKit cumulative sequence plot. The grey line shows cumulative length for all scaffolds. Coloured lines show cumulative lengths of scaffolds assigned to each phylum using the buscogenes taxrule. An interactive version of this figure is available at https://blobtoolkit.genomehubs.org/view/ilAmpBerb1.1/dataset/CAJVCG01/cumulative.

**Figure 5.**
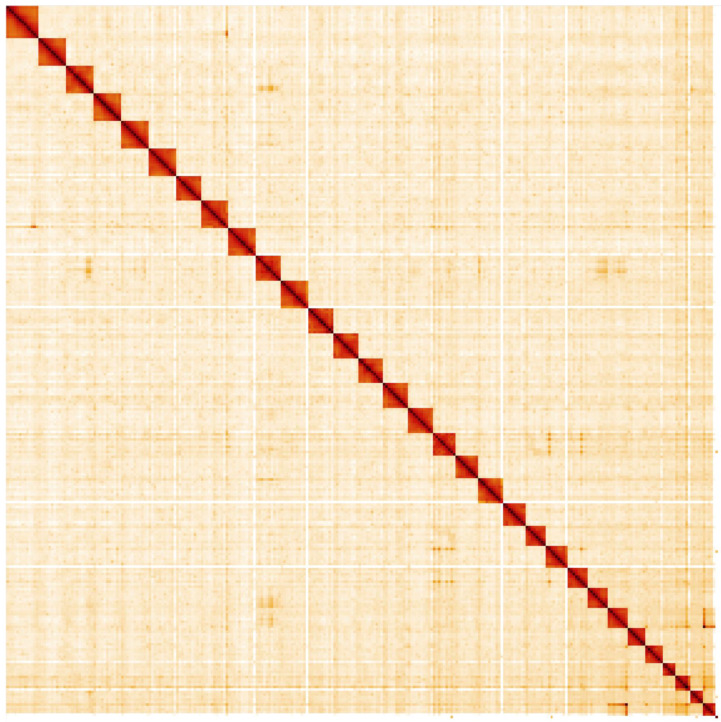
Genome assembly of *Amphipyra berbera*, ilAmpBerb1.1: Hi-C contact map. Hi-C contact map of the ilAmpBerb1.1 assembly, visualised in HiGlass.

**Table 2.** Chromosomal pseudomolecules in the genome assembly of *Amphipyra berbera*, ilAmpBerb1.1.

INSDC accession	Chromosome	Size (Mb)	GC%
OU343122.1	1	23.51	38
OU343123.1	2	22.83	38.3
OU343124.1	3	22.62	38.3
OU343125.1	4	22.01	38.5
OU343126.1	5	21.94	38.3
OU343127.1	6	21.88	38
OU343128.1	7	21.87	38.2
OU343129.1	8	21.69	38
OU343130.1	9	21.69	38.1
OU343131.1	10	21.37	38.1
OU343132.1	11	21.16	38.1
OU343133.1	12	20.95	38.3
OU343134.1	13	20.13	38.4
OU343135.1	14	20.09	38.5
OU343136.1	15	19.93	38.5
OU343137.1	16	19.30	38.7
OU343138.1	17	19.22	38.7
OU343139.1	18	19.18	38.5
OU343140.1	19	18.34	38.9
OU343141.1	20	17.80	39
OU343142.1	21	17.29	38.5
OU343143.1	22	17.23	39.1
OU343144.1	23	16.43	38.9
OU343145.1	24	15.70	39.1
OU343146.1	25	14.40	39.2
OU343147.1	26	13.64	39.1
OU343148.1	27	11.78	39.8
OU343149.1	28	11.32	40.6
OU343150.1	29	10.88	39.9
OU343151.1	30	9.24	40.2
OU343121.1	Z	26.79	37.8
OU343152.1	MT	0.02	21
-	-	0.12	48.3

## Methods

### Sample acquisition and DNA extraction

A single male *A. berbera* (ilAmpBerb1) was collected from Wytham Woods, Oxfordshire (biological vice-county: Berkshire), UK (latitude 51.772, longitude -1.338) by Douglas Boyes, UKCEH, using a light trap. The sample was identified by the same individual, and preserved on dry ice.

DNA was extracted at the Tree of Life laboratory, Wellcome Sanger Institute. The ilAmpBerb1 sample was weighed and dissected on dry ice with tissue set aside for Hi-C sequencing. Abdomen tissue was cryogenically disrupted to a fine powder using a Covaris cryoPREP Automated Dry Pulveriser, receiving multiple impacts. Fragment size analysis of 0.01-0.5 ng of DNA was then performed using an Agilent FemtoPulse. High molecular weight (HMW) DNA was extracted using the Qiagen MagAttract HMW DNA extraction kit. Low molecular weight DNA was removed from a 200-ng aliquot of extracted DNA using 0.8X AMpure XP purification kit prior to 10X Chromium sequencing; a minimum of 50 ng DNA was submitted for 10X sequencing. HMW DNA was sheared into an average fragment size between 12–20 kb in a Megaruptor 3 system with speed setting 30. Sheared DNA was purified by solid-phase reversible immobilisation using AMPure PB beads with a 1.8X ratio of beads to sample to remove the shorter fragments and concentrate the DNA sample. The concentration of the sheared and purified DNA was assessed using a Nanodrop spectrophotometer and Qubit Fluorometer and Qubit dsDNA High Sensitivity Assay kit. Fragment size distribution was evaluated by running the sample on the FemtoPulse system.

### Sequencing

Pacific Biosciences HiFi circular consensus and 10X Genomics read cloud DNA sequencing libraries were constructed according to the manufacturers’ instructions. Sequencing was performed by the Scientific Operations core at the Wellcome Sanger Institute on Pacific Biosciences SEQUEL II and Illumina HiSeq X instruments. Hi-C data were generated from head/thorax tissue using the Arima v2 Hi-C kit and sequenced on an Illumina NovaSeq 6000 instrument.

### Genome assembly

Assembly was carried out with Hifiasm ( Cheng *et al.*, 2021); haplotypic duplication was identified and removed with purge_dups ( Guan *et al.*, 2020). One round of polishing was performed by aligning 10X Genomics read data to the assembly with longranger align, calling variants with freebayes ( Garrison & Marth, 2012). The assembly was then scaffolded with Hi-C data ( Rao *et al.*, 2014) using SALSA2 ( Ghurye *et al.*, 2019). The assembly was checked for contamination and corrected using the gEVAL system ( Chow *et al.*, 2016) as described previously ( Howe *et al.*, 2021). Manual curation ( Howe *et al.*, 2021) was performed using gEVAL, HiGlass ( Kerpedjiev *et al.*, 2018) and Pretext. The mitochondrial genome was assembled using MitoHiFi ( Uliano-Silva *et al.*, 2021). The genome was analysed and BUSCO scores generated within the BlobToolKit environment ( Challis *et al.*, 2020). Table 3 contains a list of all software tool versions used, where appropriate.

**Table 3.** Software tools used.

Software tool	Version	Source
Hifiasm	0.15	( Cheng *et al.*, 2021)
purge_dups	1.2.3	Guan *et al.*, 2020
SALSA2	2.2	Ghurye *et al.*, 2019
longranger align	2.2.2	https://support.10xgenomics.com/genome-exome/ software/pipelines/latest/advanced/other-pipelines
freebayes	1.3.1-17-gaa2ace8	Garrison & Marth, 2012
MitoHiFi	2.0	( Uliano-Silva *et al.*, 2021)
gEVAL	N/A	Chow *et al.*, 2016
HiGlass	1.11.6	( Kerpedjiev *et al.*, 2018)
PretextView	0.2.x	https://github.com/wtsi-hpag/PretextView
BlobToolKit	2.6.2	Challis *et al.*, 2020

### Ethics/compliance issues

The materials that have contributed to this genome note have been supplied by a Darwin Tree of Life Partner. The submission of materials by a Darwin Tree of Life Partner is subject to the Darwin Tree of Life Project Sampling Code of Practice. By agreeing with and signing up to the Sampling Code of Practice, the Darwin Tree of Life Partner agrees they will meet the legal and ethical requirements and standards set out within this document in respect of all samples acquired for, and supplied to, the Darwin Tree of Life Project. Each transfer of samples is further undertaken according to a Research Collaboration Agreement or Material Transfer Agreement entered into by the Darwin Tree of Life Partner, Genome Research Limited (operating as the Wellcome Sanger Institute), and in some circumstances other Darwin Tree of Life collaborators.

## Data availability

European Nucleotide Archive: Amphipyra berbera (Svensson's copper underwing). Accession number PRJEB45130; https://identifiers.org/ena.embl/PRJEB45130.

The genome sequence is released openly for reuse. The *A. berbera* genome sequencing initiative is part of the Darwin Tree of Life (DToL) project. All raw sequence data and the assembly have been deposited in INSDC databases. The genome will be annotated and presented through the Ensembl pipeline at the European Bioinformatics Institute. Raw data and assembly accession identifiers are reported in Table 1.

## Author information

Members of the University of Oxford and Wytham Woods Genome Acquisition Lab are listed here: https://doi.org/10.5281/zenodo.4789929.

Members of the Darwin Tree of Life Barcoding collective are listed here: https://doi.org/10.5281/zenodo.4893704.

Members of the Wellcome Sanger Institute Tree of Life programme collective are listed here: https://doi.org/10.5281/zenodo.5377053.

Members of Wellcome Sanger Institute Scientific Operations: DNA Pipelines collective are listed here: https://doi.org/10.5281/zenodo.4790456.

Members of the Tree of Life Core Informatics collective are listed here: https://doi.org/10.5281/zenodo.5013542.

Members of the Darwin Tree of Life Consortium are listed here: https://doi.org/10.5281/zenodo.4783559.
