# Supplementary material for: The genome sequence of Svensson’s copper underwing, Amphipyra berbera Rungs, 1949
Source: Wellcome Open Res. 2021 Nov 18;6:314. [Version 1] doi: 10.12688/wellcomeopenres.17350.1 (PMC11499867; doi:10.12688/wellcomeopenres.17350.1)
Supplement: Peer review history

# Reviewer report: Reviewer response for version 1

**DOI:** 10.21956/wellcomeopenres.19180.r103652

**Published:** 2024-10-23

**Authors:** Shiqi Luo

**Affiliations:** 1 Department of Entomology, College of Plant Protection, China Agricultural University, Beijing, China

This paper presents the genome assembly of Svensson’s copper underwing at the chromosomal level. The sequencing and bioinformatics analysis methods used were sound, resulting in a high-quality assembly. 

 The paper briefly describes the genome assembly and provides useful information on how to access and download the original sequencing data and genome assembly.

 While the software tools used are listed, it would be helpful to include the main parameters utilized with these tools.

Are sufficient details of methods and materials provided to allow replication by others?

Yes

Is the rationale for creating the dataset(s) clearly described?

Yes

Are the datasets clearly presented in a useable and accessible format?

Yes

Are the protocols appropriate and is the work technically sound?

Yes

Reviewer Expertise:

insect functional genomics, non-coding RNAs

I confirm that I have read this submission and believe that I have an appropriate level of expertise to confirm that it is of an acceptable scientific standard.

# Reviewer report: Reviewer response for version 1

**DOI:** 10.21956/wellcomeopenres.19180.r103659

**Published:** 2024-10-23

**Authors:** Sivasankaran Kuppusamy

**Affiliations:** 1 Loyola College, Chennai, Tamil Nadu, India

Chromosome level genome assembly was carried out from *Amphipyra berbera* Rungs, 1949. Appropriate software was used for the assembly and annotation. The comprehensive data will be useful for the phylogenomic study of lepidopteran moths

**Comments on the manuscript:**

 The taxonomic details “(Svensson’s copper underwing; Arthropoda; Insecta; Lepidoptera; Noctuidae)” of the species need not be mentioned in the abstract.

 Mitochondrial genome size is not given in the abstract. It can be given in the abstract. 

 In the background first paragraph first sentence can be started as *Amphipyra berbera* Rungs, 1949

 Through genome assembly authors would have received the protein-coding genes and gene transcripts. The protein-coding genes and gene transcripts haven’t been included the information in the text and table. Explain.

 Mitochondrial genome sequence length 15,345 bp can be included in the text.  

 Above all, I confirm that the manuscript meets the necessary scientific standard and is suitable for indexing"

Are sufficient details of methods and materials provided to allow replication by others?

Yes

Is the rationale for creating the dataset(s) clearly described?

Yes

Are the datasets clearly presented in a useable and accessible format?

Yes

Are the protocols appropriate and is the work technically sound?

Yes

Reviewer Expertise:

Phylogenetic analysis of superfamily Noctuoidea moths using mitochondrial geneome

I confirm that I have read this submission and believe that I have an appropriate level of expertise to confirm that it is of an acceptable scientific standard.

# Reviewer report: Reviewer response for version 1

**DOI:** 10.21956/wellcomeopenres.19180.r51509

**Published:** 2022-07-27

**Authors:** Sina Beier

**Affiliations:** 1 MRC Toxicology Unit, Cambridge, UK

This study presents the chromosome level assembly of the genome of the Svenson's copper underwing.

 It was assembled following the state-of-the-art pipeline developed and used by the Darwin Tree of Life project including long and short read sequencing . This resulted in a high quality assembly which is based on the assembly of the log reads and polished with the short reads, leading to a great resolution of haplotype-resolved and nearly complete chromosomes.

 The data is presented in a concise way and leaves me to only ask for the possibility to add the W chromosome which is currently missing in a later state of the project.

Are sufficient details of methods and materials provided to allow replication by others?

Yes

Is the rationale for creating the dataset(s) clearly described?

Yes

Are the datasets clearly presented in a useable and accessible format?

Yes

Are the protocols appropriate and is the work technically sound?

Yes

Reviewer Expertise:

bioinformatics, genome assembly, genome assembly of lepidoptera

I confirm that I have read this submission and believe that I have an appropriate level of expertise to confirm that it is of an acceptable scientific standard.
